# Predictors of medicine redistribution at public healthcare facilities in King Cetshwayo District, KwaZulu-Natal, South Africa

**DOI:** 10.1186/s12913-023-10096-4

**Published:** 2023-10-17

**Authors:** Sibusiso Mabizela, Hilma N. Nakambale, Varsha Bangalee

**Affiliations:** Department of Pharmaceutical Sciences, School of Health Sciences, University of KwaZulu-Natal, Westville Campus, Durban, South Africa

**Keywords:** Stockouts, Overstocking, Expiry, National medicine policy, Inventory management, Pharmacy policy

## Abstract

**Background:**

Effective pharmaceutical inventory management is essential for optimizing healthcare outcomes and supply chain performance. However, challenges such as stockouts, overstocking, and wastage can hinder this process. This study examines the interrelationships between overstocking, stockouts, and wastage in eight healthcare facilities in Northern KwaZulu-Natal, South Africa. It also explores the extent of these challenges and investigates the use of medicine redistribution as a strategy to address inventory management issues.

**Methods:**

A retrospective quantitative analysis was conducted using pharmacy inventory records from public healthcare facilities. Eight facilities, including hospitals and a community healthcare center in King Cetshwayo District, were purposively sampled. Linear regression analysis was used to examine the association between medicine redistribution as the outcome and the predictors - stockouts, overstocking, and wastage. Pearson’s correlation was utilized to evaluate associations between the predictors. Descriptive statistics were employed to quantify the levels and extent of overstocking, stockouts, and wastage related to expiry.

**Results:**

The study included eight healthcare facilities with pharmacy warehouses managed by pharmacists. A total of 392 medicines were analyzed (49 per facility). Stockouts affected 85.6% of medicines, while overstocking and expiry-related wastage impacted 50.6% and 15.2% of medicines, respectively. The most common stock-out medicines were salbutamol 200mcg inhalant (4.0%), paracetamol 500 mg tablets (3.5%), and azithromycin 500 mg tablets (3.3%). Overstocking, stock with short-dated expiry, and expired medicines explained 68% of redistribution transactions to other facilities (r² = 0.68). A moderate, statistically significant correlation was observed between overstocking and expiry-related wastage (r² = 0.47, p-value = 0.020). Stockouts had a weak correlation with redistribution, accounting for only 4.5% (p-value < 0.01). A weak correlation was found between stockouts and overstocking (r = 0.10), as well as between stockouts and expired medicines (r = -0.20).

**Conclusion:**

This study highlights significant challenges in inventory management, particularly regarding stockouts, overstocking, and expiry-related wastage in the evaluated healthcare facilities. Medicine redistribution emerged as a viable strategy to address these challenges. Improving inventory management practices and implementing targeted interventions are crucial for optimizing pharmaceutical supply chain performance and enhancing healthcare delivery outcomes in this setting.

**Supplementary Information:**

The online version contains supplementary material available at 10.1186/s12913-023-10096-4.

## Background

Access to medicines plays a crucial role in the effective functioning of a healthcare system, as they are vital for improving healthcare outcomes in populations [1]. While the South African government has made progress in addressing challenges related to medicine accessibility by strengthening primary healthcare services across various communities, significant barriers still exist in ensuring the adequate availability and distribution of essential medicines [2]. The country continues to experience national medicine stockouts, posing a threat to the quality of healthcare services provided [3, 4]. Such stockouts are a common occurrence within the pharmaceutical supply chain system and are worsened by issues such as overstocking and wastage due to medicine expiry at healthcare facilities [3, 5–8]. These prolonged stockouts erode public trust in the healthcare system [8], leading to decreased confidence in primary healthcare clinics, as evidenced by a survey conducted in the Eastern Cape province in 2014 [2].

The World Health Organization (WHO) defines essential medicines as those that meet the priority healthcare needs of a population, selected based on disease prevalence, public health significance, evidence-based efficacy and safety, and cost-effectiveness within a specific region [9]. Thus, it is crucial that essential medicines, as listed in the essential medicines list, are consistently available in all healthcare facilities, in the correct quantities and dosage forms, to ensure optimal access for the population [9, 10]. Poor inventory management in pharmaceutical supply chain systems has severe consequences for healthcare delivery. For instance, poor inventory management practices in public sector hospitals leads to increased holding costs due to overstocking while depriving patients of necessary treatment [7]. Furthermore, inefficient inventory management practices can result in wastage due to medicine expiry when there is a lack of accurate estimation of medicine stock flow within the system [11]. Another important factor in pharmacy inventory management is the issue of maldistribution of stock, which often manifests as overstocking of certain medicines in some healthcare facilities while other facilities within the same region face stockouts of the same medicines [7]. Redistributing medicines between healthcare facilities has been proposed as an initial step in addressing inventory management challenges [12]. In Uganda, redistribution has been implemented since 2012 as a national strategy to tackle overstocking, wastage, and stockouts [5].

In South Africa, medicines and pharmaceutical supplies are procured at the national level through a centralized system, from pharmaceutical wholesalers, manufacturers, and distributors. District hospitals receive pharmaceutical supplies through a semi-decentralized system from provincial distribution warehouses, which, in turn, obtain supplies from the national warehouse where national procurement occurs. District hospital warehouses then supply hospital pharmacies, healthcare centers, and clinics. The point of care where patients receive medicines is typically at the pharmacies located within hospitals, clinics, and health centers. While stockouts and certain causes such as overstocking and expired medicines might be inevitable in specific cases, healthcare facilities can consider redistributing supplies among different facilities. This approach can help alleviate the burden and enhance medicine availability. The current extent of redistribution practices and whether inventory management challenges like stockouts, overstocking, and expired medicines encourage redistribution is not well documented in South Africa. This study aimed to investigate the influence of predictors (namely, stockouts, overstocking, and expiry-related wastage) on stock redistribution among health facilities in the King Cetshwayo District of KwaZulu-Natal, South Africa. Additionally, the study seeks to identify the medicines most affected by overstocking, stockouts, and expiry-related wastage.

## Methods

### Study design and setting

This retrospective quantitative study examined data from April 2021 to March 2022 financial year at health facilities in King Cetshwayo District, KwaZulu-Natal province, South Africa. The district spans 8,213 square kilometers and is divided into Mfolozi, Mhlathuze, Mlalazi, Mthonjaneni, and Nkandla local municipalities [13]. The district includes one tertiary hospital, one regional hospital, six district hospitals, one community health center, 63 primary healthcare clinics, and 16 mobile clinics. These public-sector healthcare facilities serve a population of over 971,135, according to the 2016 census [14].

### Sample size and selection criteria

A purposive sampling method was employed to select nine healthcare facilities based on the population they serve, which included public sector hospitals and a community healthcare center in King Cetshwayo District [15]. Primary healthcare clinics were excluded from the study due to their reliance on manual pharmacy information management systems, such as paper stock cards. Additionally, public healthcare facilities that declined participation were also excluded. For confidentiality purposes, facilities were represented with a code consisting of the word ‘FAC’ short for facility, and an ID number starting from one (example: FAC-001, represents facility number one), (Supplementary: Table S1). A total of 49 medicines were selected from the Essential Medicines List and Standard Treatment Guidelines Hospital Level Adult – 2019 Edition and Paediatric – 2017 [16]. The medicines were randomly chosen using the randomization function in Microsoft Excel. These medicines were assessed across eight healthcare facilities which responded to the survey, resulting in a total of 392 medicines, (Supplementary: Table S2).

### Data collection

Quantitative data were extracted from the electronic pharmacy information management system (ePIMS) known as RxSolution®. The community healthcare centers and hospitals in King Cetshwayo District utilize ePIMS to manage pharmacy inventory data, including details on medicines, orders, storage, and distribution transactions. The system encompasses transactions and reports modules that summarize inventory data. Data for this study were collected by generating stock movement and receipt reports from the reports module, which provided a summary of stockouts, overstocking, expired medicines, and short-dated expiry [17, 18].

For the purpose of this study, the following terms were defined as follows: Stockout/s - refers to the complete unavailability of a specified medicine formulation or dosage for a period of one day or more at a specified health facility [17]; overstocking - refers to keeping amounts of medicines in excess of three months of consumption; expiry-related wastage - refers to unusable medicines that have been kept on pharmacy shelves beyond their expiry date, resulting in obsolescence and disposal [18]; redistribution - refers to the distribution of medicines between health facilities to meet demand and improve access without the involvement of primary suppliers.

### Data Analysis

Prior to conducting analyses, the main variables of the study were defined to measure specific objectives. These variables included stockouts (measured by frequency and duration), overstocking (measured by quantity in units), expiry-related wastage (measured by stock received with short-dated expiry, expired medicines in units, and the value of expired medicines), and redistribution (measured by stock received from and issued to other healthcare facilities). The data were extracted from RxSolution® and cleaned using Microsoft Excel spreadsheet. Variables were analyzed using IBM SPSS® windows, version 28.

A two-tailed Pearson’s correlation analysis was conducted to examine the potential correlation between stockouts, overstocking, and expiry-related wastage. Additionally, multiple linear regression analyses were performed to determine if pharmacy inventory management challenges (overstocking, stockouts, and expiry-related wastage) could predict redistribution at public healthcare facilities.

In the first analysis, the frequency of stockouts was tested as a predictor of redistribution, specifically stock received from other facilities. In the second analysis, linear regression was utilized to investigate whether overstocking, stock received with a short-dated expiry date, and expired medicines were significant predictors of redistribution, with redistribution (stock issued to other facilities) as the outcome variable. Any missing values were excluded from the analysis.

To ensure a more normal distribution of the data and enhance the validity of parametric tests, log transformation was applied to the data prior to statistical analysis. Log transformation was achieved by taking the common logarithm (log10) of the raw data. All statistical tests were performed on the log-transformed data, and the results are reported in terms of the log-transformed values. To facilitate the interpretation and communication of the results, the estimates were back-transformed by taking the exponent of the log-transformed values (i.e., 10 raised to the power of the log-transformed value).

## Results

### Healthcare facility selection and eligibility

**Fig. 1:**
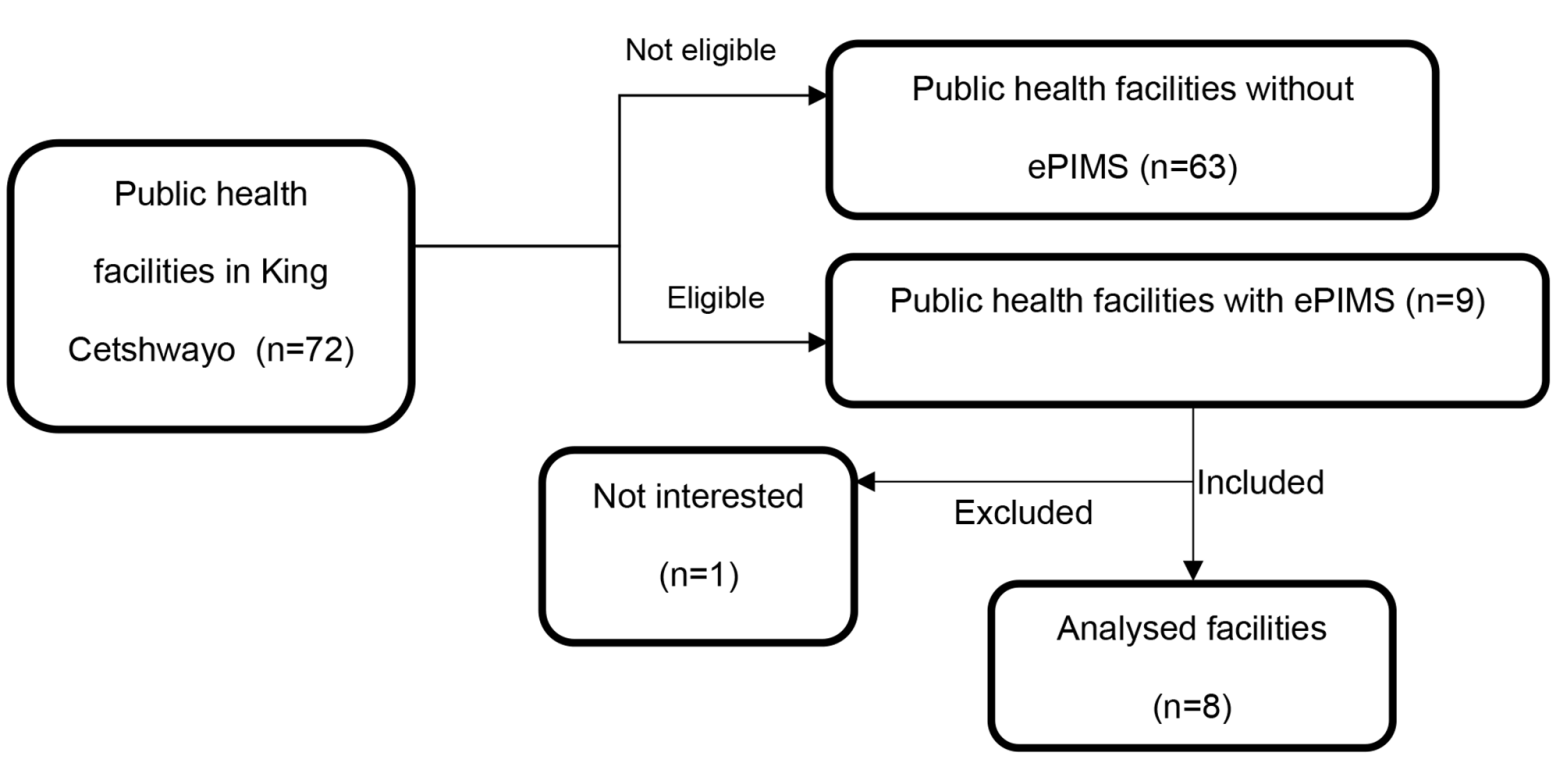
Flowchart of facility inclusion and exclusion

A total of 63 facilities, primary health care clinics were excluded because they manual records for inventory management. Out of the nine eligible public healthcare facilities – which utilize ePMS for stock management, one tertiary hospital declined to participate, resulting in a response rate of 88.89% (Fig. 1). The analyzed public health facilities comprised of one community healthcare center (12.5%), six district hospitals (75%), and one regional hospital (12.5%). All pharmacy stores/warehouses in these public healthcare facilities were under the management of pharmacists. A total of 49 medicines chosen per facility for the study, multiplied by the eight facilities that participated in the study, totalled to 392 medicine inventory items for the study.

### Levels of pharmacy inventory management challenges

#### Stockouts

Stockouts had a substantial impact on 334 (85.20%) medicines across the eight public healthcare facilities in King Cetshwayo District. On average, there were 2.3 stockouts per month (SD = 1.9), with an average duration of 22.4 days (SD = 3.1). District hospitals experienced more frequent and longer-lasting stockouts, with FAC-003 recording the highest average monthly number of stockouts (M = 3.1, SD = 2.2) and the longest average duration (M = 35.5, SD = 3.2) among all facilities. The most commonly stockout medicines were salbutamol 100mcg inhalant (4.0%), paracetamol 500 mg tablets (3.5%), and azithromycin 500 mg tablets (3.3%). Diazepam 10 mg/2ml injection had the highest average number of stockouts (M = 47.3, SD = 2.3).

### Overstocking

The findings indicated that overstocking affected 197 (50.26%) of the medicine inventory in King Cetshwayo District across the eight healthcare facilities. FAC-004 had the highest average overstocking (M = 2.6, SD = 0.7), while FAC-007 had the lowest average (M = 1.6, SD = 1.0). Tenofovir, lamivudine & dolutegravir 50,300&300 mg tablets were the most overstocked item (M = 4.1, SD = 0.3), while methylphenidate 10 mg tablets were the least overstocked item (M = 0.4, SD = 0.4).

### Expired medicines

The analysis showed that the issue of expired medicines impacted 61 (15.56%) of the medicines across the eight healthcare facilities in King Cetshwayo District. Among the facilities, FAC-007 had the highest average value of expired medicines (M = 3.2, SD = 0.9), while FAC-002 had the lowest average value (M = 2.5, SD = 0.6). Adrenaline 1 mg/ml injection was the most frequently expired medicine, (M = 1.56, SD = 0.43) whereas ibuprofen 200 mg tables were the least expired (M = 0.48, SD = 0.35).

### Association between pharmacy inventory management challenges

Pearson’s correlation analysis was performed to explore the relationships between various pharmacy inventory management challenges, including stockouts (frequency and duration in days), overstocking, stock received with short-dated expiry, expired medicines, and the value of expired medicines, (Table 1**)**. A significant positive correlation was observed between overstocking and expired medicines (r = 0.47, p-value < 0.05), as well as between overstocking and stock received with short-dated expiry (r = 0.40, p-value < 0.01). Furthermore, stock received with short-dated expiry showed a significant correlation with expired medicines (r = 0.66, p-value < 0.01). The results revealed a weak correlation between stockouts (frequency) and overstocking (r = 0.10), as well as between stockouts (frequency) and expired medicines (r = -0.20).

**Table 1:** Pearson correlation matrix for pharmacy inventory management challenges

Variables	Stockouts (Frequency)	Stockouts (Period)	Overstocking (Units)	Stock received with short-dated expiry (Units)	Expired medicines (Units)	Value of expired medicines
Stockouts (Frequency)						
Stockouts (Period)	0.72**					
Overstocking (Units)	0.10	-0.10				
Stock received with short-dated expiry (Units)	0.06	-0.13	0.40**			
Expired medicines (Units)	-0.20	-0.04	0.47*	0.66**		
Value of expired medicines	-0.09	-0.01	0.26	0.59**	0.82**	

*Note.* **Correlation significant at *p* < 0.01 (2-tailed), *Correlation significant at *p* < 0.05 (2-tailed)

### Pharmacy inventory management challenges and redistribution strategy

**Fig. 2:**
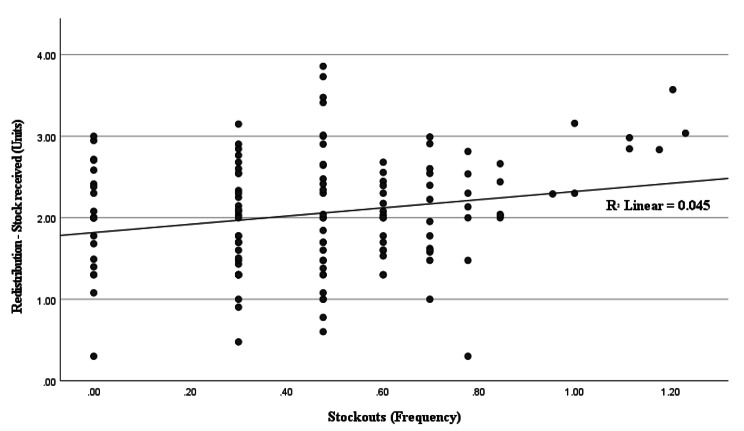
Scatterplot for the linear relationship between stockouts frequency and redistribution – stock received

To investigate the relationship between pharmacy inventory management challenges and the redistribution strategy, a linear regression analysis was performed. The study found that the frequency of stock shortages only explained 4.5% of the variation in supplies received from other healthcare facilities (r^2^ = 0.045, p-value = 0.012) (Fig. 2).

In a multivariate regression analysis, the predictors (overstocking, stock received with short-dated expiry, and expired medicines) explained 68% of the redistribution transactions issued to other healthcare facilities (r^2^ = 0.68, p-value < 0.001). Both overstocking (β = 0.39, p-value = 0.004) and stock received with short-dated expiry (β = 0.41, p-value = 0.022) were statistically significant predictors of redistribution, while expired medicines did not significantly predict redistribution (β = 0.08, p-value = 0.574). A subsequent regression analysis, which excluded expired medicines and focused only on overstocking and stock received with short-dated expiry, did not significantly affect the collective multiple linear regression coefficient mentioned above (r^2^ = 0.68). Thus, it can be inferred that overstocking and stock received with short-dated expiry were the main contributors to the redistribution of stock to other healthcare facilities. These two variables, overstocking and stock received with short-dated expiry, explained 67% of the variance in stock issued to other healthcare facilities, exhibiting a statistically significant collective effect (r^2^ = 0.67, p-value < 0.001). Specifically, overstocking (β = 0.41, p-value < 0.001) and stock received with short-dated expiry (β = 0.46, p-value < 0.001) significantly predicted the stock issued to other healthcare facilities.

**Fig. 3:**
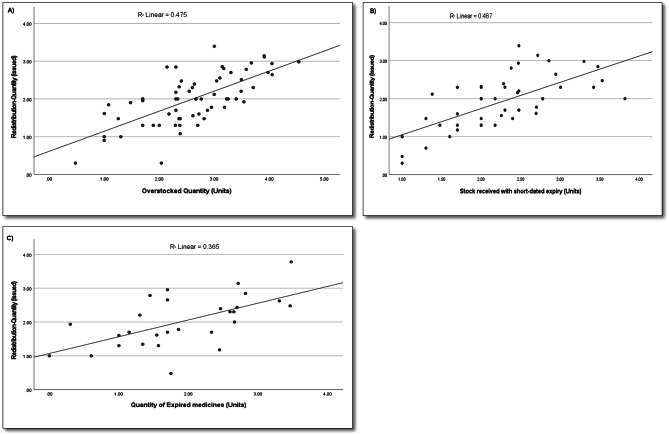
Scatterplots for correlations between predictor variables (overstocking, stock received with short-dated expiry, and expiry- related wastage) and redistribution, stock issued to other facilities

**Figure – was 3 (A)** shows a scatterplot illustrating the linear correlation between overstocking and redistribution, stock issued to other facilities (r^2^ = 0.475, p-value < 0.001). Figure 3**(B)** shows a scatterplot demonstrating the linear correlation between stock received with short-dated expiry and redistribution, stock issued to other facilities (r^2^ = 0.467, p-value < 0.001). Finally, Fig. 3**(C)** shows a scatterplot illustrating the linear relationship between expired medicines and redistribution, stock issued to other facilities (r^2^ = 0.365, p-value < 0.001).

## Discussion

Effective pharmacy inventory management is crucial for ensuring high-quality healthcare services [1]. However, challenges such as stockouts, overstocking, and expiry-related wastage are prevalent in many healthcare facilities in South Africa. This study aimed to evaluate the correlations, prevalence, and impact of medicine stockouts, overstocking, and expiry-related wastage in eight healthcare facilities in King Cetshwayo District, Northern KwaZulu-Natal, South Africa. The findings underscore the need to improve inventory management at the healthcare facility level and standardize medicine redistribution to reduce wastage, overstocking, and stockouts.

The most prevalent inventory management challenge identified in all eight healthcare facilities was stockouts, which occurred with varying durations. Essential medicines like salbutamol 200mcg inhalant, paracetamol 500 mg tablets, and azithromycin 500 mg tablets were frequently stocked out, posing a threat to the continuity of pharmaceutical care and increasing the risk of treatment failure [7]. Previous studies on medicine stockouts in South Africa have confirmed that stockouts are a national crisis [3, 19]. Implementing a well-integrated inventory management system has proven challenging at both the provincial and national levels in South Africa in recent years. In addition to such a system, training pharmacy personnel in inventory management is essential for improving the overall supply chain system. Thus, a concerted effort is required from all pharmaceutical supply chain stakeholders, starting from the national level, to address persistent inventory management challenges [12, 20, 21].

Understanding the correlations between pharmacy inventory management challenges is crucial for enhancing the supply chain system. Previous studies have highlighted the complexities in determining the causes of medicine stockouts [20, 22–26]. The findings of this study indicate no statistically significant correlation between stockouts and either overstocking or expiry-related wastage. This reflects the intricacies involved in determining the causes of stockouts at the facility level, which may explain why facilities continue to experience stockouts. The failure to identify the root causes of stockouts makes it challenging to implement effective measures to address this challenge. However, it should be noted that the accuracy of the data may have influenced the findings, as record-keeping remains poor in most healthcare facilities in South Africa [4, 27].

A significant correlation was observed between overstocking and expiry-related wastage. Overstocking increases government spending on medicines and contributes to medicine wastage [9]. Improving inventory management practices, including training pharmacy staff and implementing an efficient inventory surveillance system, can help reduce medicine wastage and save costs. The study also revealed that expiry-related wastage of medicine was primarily attributed to stock received with a short expiry date. Similar results have been found in other studies, highlighting that stock received with a short expiry date and overstocking are leading causes of expiry-related wastage [11, 28]. The delivery of medicines with a short expiry date could be attributed to poor coordination within the supply chain system, resulting in delayed delivery of medicines to healthcare facilities [30]. Although this study did not assess the time-to-expiry after the medicines were delivered at healthcare facilities, a short expiry date upon delivery may have contributed to expiration-related wastage and stockouts. Documentation and record-keeping are crucial to ensure that all supply chain stakeholders are aware of short-expiry-dated pharmaceutical products, allowing for their prompt prioritized consumption before expiry.

Stockouts occur when inventory levels are insufficient to meet demand, leading to compromised patient care and treatment outcomes. Conversely, overstocking can result in higher carrying costs and the potential for medications to expire before their use. Expired medications not only contribute to significant financial losses but also pose environmental waste management challenges. To tackle these issues, pharmacy managers can take proactive measures, such as implementing accurate forecasting methods, cultivating strong relationships with suppliers, and establishing efficient inventory management systems. One effective strategy is the adoption of a first-in-first-out (FIFO) method, which minimizes the risk of medicine expiry. Additionally, monitoring expiration dates and implementing a robust inventory rotation strategy can help reduce the number of wasted medicines.

Successful pharmacy inventory management necessitates careful planning and decision-making [11, 29]. The challenges of stockouts, overstocking, and expiry-related wastage identified in this study are primarily rooted in the maldistribution of medicines and poorly coordinated inventory management. Maldistribution of medicines undermines healthcare service delivery and increase morbidity. Addressing inventory management challenges requires a systematic approach to prevent stockouts, overstocking, and wastage. Stakeholders at multiple levels, from the national to the provincial level, must collaborate to implement well-coordinated and real-time (synchronized) inventory management surveillance tools. The synchronized redistribution system will ensure that pharmacy providers are able to see stock levels at other facilities, thus will enable effective channelling of short expiry dated medicines to healthcare facilities with high consumption rates.

Redistribution does not only reduce wastage, it also ensures the availability of medicines when and where they are needed. Lessons can be drawn from successful implementation of redistribution strategies in countries like Uganda, where redistribution has been a national strategy since 2012, supported by dedicated budgets and guidelines to improve medicine circulation and accessibility [5]. A study conducted in Saudi Arabia also recommended adopting a centralized inventory management approach to promote equitable distribution of medicines and enhance accessibility [12]. To establish an integrated inventory management system, a robust pharmacy information system is crucial for ensuring real-time stock availability surveillance across provinces. For a centralized redistribution system to be successful, comprehensive training for pharmaceutical supply chain staff is also paramount to enable effeciency.

Improving inventory management at all levels of the public sector healthcare system requires systemic solutions and collaborative efforts from multiple stakeholders. These efforts should include identifying and defining specific indicators for optimal inventory management as part of the national medicine policy [21]. The existing electronic Pharmacy Information Management System (ePIMS) used in public-sector healthcare facilities in South Africa needs updating to incorporate an automated alarm system for tracking medicine expiry dates, overstocking, and understocking. Additionally, expanding the integrated ePIMS to primary healthcare clinics would enhance medicine availability surveillance. Policymakers must introduce official guidelines for medicine redistribution as an integrated inventory management strategy to improve medicine accessibility within the healthcare system. Future research should aim to explore the associations between inventory management challenges, and compliance with public sector financial management principles during redistribution.

### Strengths and limitations of the study

Our study has several limitations that should be acknowledged. Firstly, the generalizability of our findings is restricted to public healthcare facilities employing the current electronic Pharmaceutical Inventory Management System (ePIMS). Thus, a separate investigation is needed to examine inventory management challenges faced by primary healthcare clinics employing manual pharmacy information management systems, such as paper stock cards. Secondly, the quality of our study may have been compromised due to the absence of updates and customization of the ePIMS to reflect current practices. Notably, the ePIMS lacks a redistribution module that adequately aligns redistribution transactions and data, potentially affecting the accuracy of our findings. Lastly, the informal and non-standardized nature of redistribution practices between health facilities might have resulted in underestimation of redistribution transactions, as some transactions may have gone unrecorded. Nonetheless, our study contributes critical insights into the issues of stockouts, overstocking, and expiry-related medicine wastage as significant inventory management challenges within the South African context. The findings highlighted specific challenges faced by public healthcare facilities in the King Cetshwayo District and can inform strategies and interventions to improve pharmaceutical inventory management in this region and similar settings.

## Conclusion

Our study reveals several key findings that contribute to the understanding of inventory management in South Africa. Firstly, we have established a strong positive correlation between overstocking and the presence of expired and short-dated medicines. This underscores the importance of implementing better inventory control measures to prevent wastage resulting from excessive stock levels. Secondly, stockouts have had a substantial impact on medicine availability, particularly in district hospitals where stockouts have been more frequent and longer lasting compared to other healthcare centers. We have identified specific medicines as commonly experiencing stockouts, which provides valuable information for targeted interventions. Thirdly, the issue of expired medicines has been prevalent, affecting a significant proportion of the medicine inventory. Variations in the average value of expired medicines across facilities suggest differences in expiration management practices. Addressing this issue requires improved expiration tracking systems and timely disposal of expired medicines. Our analyses have also shown that stockouts significantly predict the stock received from other healthcare facilities. Lastly, overstocking and stock received with short-dated expiry emerged as significant predictors of stock redistribution. These findings provide valuable insights for policymakers, managers, and practitioners to develop targeted interventions and policies aimed at improving pharmaceutical inventory management in South Africa.

Overall, this study highlights the urgent need for enhanced pharmaceutical inventory management practices in public healthcare facilities. Strategies should focus on reducing overstocking, preventing stockouts, and improving expiration management. Strengthening collaboration and communication among healthcare facilities to facilitate effective redistribution can contribute to addressing inventory management challenges.

### Electronic supplementary material

Below is the link to the electronic supplementary material.

Supplementary Material 1

## Data Availability

All the data collected are available in this manuscript and supplementary material.

## List of abbreviations

WHO: World Health Organization
ePMIS: electronic Pharmaceutical Inventory Management System
FIFO: first-in-first-out
