# Supplementary material for: Predictors of medicine redistribution at public healthcare facilities in King Cetshwayo District, KwaZulu-Natal, South Africa
Source: BMC Health Serv Res. 2023 Oct 17;23:1108. doi: 10.1186/s12913-023-10096-4 (PMC10583440; doi:10.1186/s12913-023-10096-4)

**Supplementary Material**

***Table S1:*** *Eligible public healthcare facilities*

| **Health facility ID** | **Facility classification** |
| --- | --- |
| FAC-001 | District hospital ^a^ |
| FAC-002 | District hospital ^a^ |
| FAC-003 | District hospital ^a^ |
| FAC-004 | District hospital ^a^ |
| FAC-005 | District hospital ^a^ |
| FAC-006 | District hospital ^a^ |
| FAC-007 | Community healthcare centre ^b^ |
| FAC-008 | Regional hospital ^c^ |
| FAC-009 | Tertiary hospital ^d^ |

*Note.* FAC = facility, FAC-001 to 009 represent IDs for eligible facilities for the study.

^a^ District hospitals are first-level health facilities that provide non-specialised healthcare to local communities. Patients may be referred from lower levels, i.e., primary healthcare clinics and community healthcare centres ^30^.

^b^ Community healthcare centre is a facility that is open 24/7 and offers a full range of primary healthcare services, such as a 24-hour emergency room and maternity and obstetric healthcare services ^30^.

^c^  Regional hospital is a second-level health facility that provides specialised healthcare to the regional community. Patients may be referred from first-level i.e., district hospitals ^30^.

^d^ Tertiary hospital is a health facility that provides more specialised healthcare services to patients referred from regional and district hospitals ^30^.

**APPENDIX 2**

**Table S2:** Medicines and anatomical therapeutic class (ATC) codes

| **Medicine class** | **Active Ingredients** | **Size/ Volume** | **ATC Codes** |
| --- | --- | --- | --- |
| Antibiotic | Amoxycillin 125mg/5ml suspension | 100ml | J01CA04 |
| Antibiotic | Azithromycin 500mg tablets | 3 | J01FA10 |
| Antibiotic | Sulfamethoxazole & trimethoprim 480mg tablets | 56 | J01EE01 |
| Antibiotic | Ceftriaxone 1000mg injection | 10ml | J01DD04 |
| Antibiotic | Flucloxacillin 250mg capsules | 40 | J01CF05 |
| Antibiotic | Metronidazole 400mg tablets | 14 | J01XD01 |
| Cardiovascular system agent | Adrenaline 1mg/ml injection | 2ml | C01CA24 |
| Blood & blood forming organs agent | Acetylsalicylic acid 300mg tablets | 14 | B01AC06 |
| Cardiovascular system agent | Enalapril 10mg tablets | 28 | C09AA02 |
| Cardiovascular system agent | Furosemide 40mg tablets | 28 | C03CA01 |
| Cardiovascular system agent | Hydrochlorothiazide 12.5mg tablets | 28 | C03AA03 |
| Cardiovascular system agent | Methyldopa 250mg tablets | 56 | C02AB02 |
| Nervous system agent | Amitriptyline 25mg tablets | 28 | N06AA09 |
| Nervous system agent | Chlorpromazine 25mg tablets | 56 | N05AA01 |
| Nervous system agent | Citalopram 20mg capsules | 28 | N06AB04 |
| Nervous system agent | Clonazepam 0.5mg tablets | 84 | N03AE01 |
| Nervous system agent | Diazepam 5mg/ml injection | 2ml | N05BA01 |
| Nervous system agent | Methylphenidate 10mg tablets | 30 | N06BA04 |
| Nervous system agent | Risperidone 3mg tablets | 30 | N05AX08 |
| Alimentary tract & metabolism agent | Glimepiride 1mg tablets | 30 | A10BB12 |
| Alimentary tract & metabolism agent | Metformin 500mg tablets | 56 | A10BA02 |
| Cardiovascular system agent | Simvastatin 10mg tablets | 28 | C10AA01 |
| Analgesic agent | Ibuprofen 200mg tablets | 15 | M01AE01 |
| Analgesic agent | Paracetamol 120mg/5ml syrup | 100ml | N02BE01 |
| Analgesic agent | Paracetamol 500mg tablets | 20 | N02BE01 |
| Analgesic agent | Pethidine 50mg/ml injection | 1ml | N02AB02 |
| Analgesic agent | Tramadol 50mg capsules | 100 | N02AX02 |
| Antiretroviral agent | Abacavir & Lamivudine 600&300mg tablets | 28 | J05AR02 |
| Antiretroviral agent | Efavirenz 200mg capsules | 84 | J05AG03 |
| Antiretroviral agent | Tenofovir,lamivudine&dolutegravir 300,300&50mg tablets | 28 | J05AR27 |
| Anti-tuberculosis agent | Ethambutol 400mg tablets | 56 | J04AM02 |
| Anti-tuberculosis agent | Isoniazid 300mg tablets | 28 | J04AC01 |
| Anti-tuberculosis agent | Rifampicin & Isoniazid 300&150mg tablets | 56 | J04AK02 |
| Alimentary tract & metabolism agent | Hyoscine 10mg tablets | 10 | A03BA03 |
| Alimentary tract & metabolism agent | Oral rehydration salt solution powder | Sachet | A07CA |
| Dermatological agent | Aqueous cream | 100g | D11AX |
| Nervous system agent | Carbamazepine 200mg tablets | 56 | N03AF01 |
| Nervous system agent | Phenobarbitone 30mg tablets | 28 | N03AA02 |
| Nervous system agent | Phenytoin 100mg capsules | 84 | N03AB02 |
| Intravenous fluids | Dextrose 5% infusion | 1000ml | V06DC01 |
| Intravenous fluids | Sodium chloride 0.9% infusion | 1000ml | V06DC01 |
| Corticosteroids | Prednisone 5mg tablets | 28 | H02AB07 |
| Respiratory system agent | Salbutamol 100mcg inhalant | 200 Doses | R03CC02 |
| Antihistamine | Chlorpheniramine 4mg tablets | 10 | R06AB04 |
| Blood & blood forming organs agent | Ferrous sulphate 200mg tablets | 28 | B03AA07 |
| Blood & blood forming organs agent | Folic acid 5mg tablets | 28 | B03BB01 |
| Alimentary tract & metabolism agent | Pyridoxine 25mg tablets | 28 | A11HA02 |
| Vaccines | Rotavirus vaccine drops | 1.5ml | J07BH01 |
| Vaccines | Tetanus toxoid vaccine injection | 10ml | J07AM01 |

***Table S3:*** *Descriptive statistics for main study variables*

| **Variables** | Log-transformed | | Back-transformed | |
| --- | --- | --- | --- | --- |
|  | Mean | Standard Deviation | Geometric Mean | Standard Deviation |
| Stockouts (frequency) (*N* = 334) | 0.4 | 0.3 | 2,3 | 1,9 |
| Stockouts (period in days) (*N* =334) | 1.4 | 0.5 | 22,4 | 3,1 |
| Overstocking (units) (*N* = 197) | 2.4 | 0.8 | 230 | 6,6 |
| Stock received with short-dated expiry (units) (*N* = 128) | 2.1 | 0.7 | 115,7 | 5,2 |
| Expired medicines (units) (*N* = 61) | 1.7 | 0.8 | 47,1 | 7 |
| Value of expired medicines (ZAR) (*N* = 61) | 2.8 | 0.9 | 657,3 | 7,4 |
| Redistribution – Stock issued (units) (*N* = 119) | 2.0 | 0.7 | 95,5 | 4,8 |
| Redistribution – Stock received (units) (*N* = 149) | 2.1 | 0.7 | 120,3 | 4,9 |

*Note.* ZAR = South African Rand.

**Table S4.** Average stockouts (frequency and period) by facility

| Facility | Stockouts (frequency) log-transformed | | Stockouts (period) log-transformed | | Stockouts (frequency) back transformed | | Stockouts (period) back transformed | |
| --- | --- | --- | --- | --- | --- | --- | --- | --- |
|  | Mean | Standard Deviation | Mean | Standard Deviation | Mean | Standard Deviation | Mean | Standard Deviation |
| FAC-001 | 0,3 | 0,3 | 1,5 | 0,5 | 2,1 | 2,0 | 32,5 | 3,1 |
| FAC-002 | 0,5 | 0,3 | 1,6 | 0,4 | 2,9 | 1,8 | 35,5 | 2,8 |
| FAC-003 | 0,5 | 0,3 | 1,6 | 0,5 | 3,1 | 2,2 | 35,5 | 3,2 |
| FAC-004 | 0,3 | 0,2 | 1,3 | 0,5 | 2,1 | 1,8 | 18,1 | 3,0 |
| FAC-005 | 0,3 | 0,3 | 1,3 | 0,3 | 2,1 | 1,9 | 19,4 | 2,2 |
| FAC-006 | 0,3 | 0,2 | 1,2 | 0,4 | 2,0 | 1,8 | 17,5 | 2,5 |
| FAC-007 | 0,4 | 0,3 | 1,3 | 0,5 | 2,4 | 1,8 | 18,8 | 2,9 |
| FAC-008 | 0,3 | 0,3 | 1,0 | 0,5 | 1,9 | 1,8 | 9,5 | 3,3 |

**Table S5.** Percentage stockouts per medicine

| **Medicine name** | **Stockouts (%)** | **Medicine name** | **Stockouts (%)** |
| --- | --- | --- | --- |
| Abacavir & lamivudine 600&300mg tablets | 1.5% | Hyoscine 10mg tablets | 2.1% |
| Adrenaline 1mg/ml injection | 2.3% | Ibuprofen 200mg tablets | 0.5% |
| Amitriptyline 25mg tablets | 1.8% | Isoniazid 300mg tablets | 1.6% |
| Amoxycillin 125mg/5ml suspension | 2.5% | Metformin 500mg tablets | 1.2% |
| Aqueous 100g cream | 1.7% | Methyldopa 250mg tablets | 2.0% |
| Acetylsalicylic acid 300mg | 1.2% | Methylphenidate 10mg tablets | 2.6% |
| Azithromycin 500mg tablet | 3.3% | Metronidazole 400mg tablets | 1.7% |
| Carbamazepine 200mg tablet | 2.1% | Oral rehydration salt solution powder | 2.5% |
| Ceftriaxone 1g | 3.1% | Paracetamol 120mg/5ml syrup | 2.4% |
| Chlorpheniramine 4mg tablet 10s | 1.7% | Paracetamol 500mg tablets | 3.5% |
| Chlorpromazine 25mg tablet 56s | 2.4% | Pethidine 50mg/ml injection | 2.2% |
| Citalopram 20mg capsules | 1.3% | Phenobarbitone 30mg tablets | 2.6% |
| Clonazepam 0,5mg tablets | 2.3% | Phenytoin 100mg tablets | 2.3% |
| Sulfamethoxazole & trimethoprim 480mg tablets | 2.1% | Prednisone 5mg tablets | 3.2% |
| Dextrose 5% infusion | 2.9% | Pyridoxine 25mg tablets | 1.1% |
| Diazepam 5mg/ml injection | 1.4% | Rifampicin & Isoniazid 300&150mg tablets | 1.1% |
| Efavirenz 200mg capsules | 1.6% | Risperidone 3mg tablets | 1.7% |
| Enalapril 10mg tablets | 2.2% | Rotavirus vaccine drops | 2.6% |
| Ethambutol 400mg tablets | 1.8% | Salbutamol 100mcg inhalant | 4.0% |
| Ferrous sulphate 2000mg tablets | 1.5% | Simvastatin 10mg tablets | 2.1% |
| Flucloxacillin 250mg capsules | 0.8% | Sodium chloride 0,9% infusion | 1.7% |
| Folic acid 5mg tablets | 2.2% | Tetanus vaccine injection | 1.8% |
| Furosemide 40mg tablets | 1.5% | Tenofovir, lamivudine & dolutegravir 50&300&300mg tablets | 3.2% |
| Glimepiride 1mg tablets | 2.2% | Tramadol 50mg tablets | 2.3% |
| Hydrochlorothiazide 12,5mg tablets | 0.6% |  |  |

**Table S6.** Stockouts (period) by medicines

| Medicines | Stockouts (period) log-transformed | | Stockouts (period) back transformed | |
| --- | --- | --- | --- | --- |
|  | Mean | Standard Deviation | Geometric Mean | Standard Deviation |
| Abacavir & Lamivudine 600/300mg tablets | 1,3 | 0,7 | 22,2 | 4,7 |
| Adrenaline 1mg/ml injection | 1,6 | 0,6 | 40,4 | 3,6 |
| Amitriptyline 25mg tablets | 1,3 | 0,6 | 22,1 | 3,6 |
| Amoxycillin 125mg/5ml suspension | 1,5 | 0,3 | 32,4 | 1,9 |
| Aqueous 100g cream | 1,4 | 0,4 | 24,2 | 2,6 |
| Acetylsalicylic acid 300mg tablets | 1,1 | 0,4 | 13,5 | 2,7 |
| Azithromycin 500mg tablets | 1,6 | 0,4 | 40,5 | 2,6 |
| Carbamazepine 200mg tablet | 1,5 | 0,4 | 31,6 | 2,4 |
| Ceftriaxone 1g injection | 1,5 | 0,5 | 28,5 | 3,3 |
| Chlorpheniramine 4mg tablets | 1,4 | 0,4 | 26,1 | 2,5 |
| Chlorpromazine 25mg tablets | 1,6 | 0,4 | 36,3 | 2,7 |
| Citalopram 20mg capsules | 1,5 | 0,5 | 28,6 | 3,0 |
| Clonazepam 0,5mg tablets | 1,4 | 0,6 | 23,4 | 3,6 |
| Sulfamethoxazole & trimethoprim 480mg tablets | 1,1 | 0,4 | 12,8 | 2,8 |
| Dextrose 5% infusion | 1,3 | 0,6 | 18,2 | 4,0 |
| Diazepam 5mg/ml injection | 1,7 | 0,4 | 47,3 | 2,3 |
| Efavirenz 200mg capsules | 1,3 | 0,5 | 21,9 | 3,3 |
| Enalapril 10mg tablets | 1,3 | 0,4 | 21,8 | 2,3 |
| Ethambutol 400mg tablets | 1,5 | 0,4 | 28,8 | 2,6 |
| Ferrous sulphate 200mg tablets | 1,4 | 0,5 | 25,1 | 2,9 |
| Flucloxacillin 250mg tablets | 1,0 | 0,3 | 9,3 | 2,0 |
| Folic acid 5mg tablets | 1,5 | 0,4 | 29,1 | 2,7 |
| Furosemide 40mg tablets | 1,1 | 0,6 | 13,1 | 3,8 |
| Glimepiride 1mg tablets | 1,3 | 0,3 | 21,6 | 2,1 |
| Hydrochlorothiazide 12,5mg tablets | 0,6 | 0,3 | 4,1 | 1,9 |
| Hyoscine 10mg tablets | 1,4 | 0,5 | 28,0 | 2,9 |
| Ibuprofen 200mg tablets | 0,5 | 0,3 | 3,0 | 2,2 |
| Isoniazid 300mg tablets | 1,2 | 0,3 | 15,2 | 2,2 |
| Metformin 500mg tablets | 0,8 | 0,7 | 6,4 | 4,8 |
| Methyldopa 250mg tablets | 1,3 | 0,5 | 19,2 | 3,1 |
| Methylphenidate 10mg tablets | 1,5 | 0,5 | 28,5 | 3,2 |
| Metronidazole 400mg tablets | 1,1 | 0,4 | 12,2 | 2,6 |
| Oral rehydration salt powder | 1,4 | 0,6 | 26,5 | 3,6 |
| Paracetamol 120mg/5ml syrup | 1,6 | 0,7 | 39,9 | 4,8 |
| Paracetamol 500mg tablets | 1,3 | 0,6 | 21,8 | 3,9 |
| Pethidine 50mg/ml injection | 1,6 | 0,4 | 42,8 | 2,2 |
| Phenobarbitone 30mg tablets | 1,5 | 0,3 | 31,9 | 2,2 |
| Phenytoin 100mg capsules | 1,4 | 0,5 | 23,3 | 2,9 |
| Prednisone 5mg tablets | 1,3 | 0,6 | 21,0 | 4,0 |
| Pyridoxine 25mg tablets | 1,0 | 0,7 | 10,8 | 5,0 |
| Rifampicin & isoniazid 300&150mg tablets | 1,2 | 0,4 | 17,1 | 2,5 |
| Risperidone 3mg tablets | 1,4 | 0,4 | 22,8 | 2,4 |
| Rotavirus vaccine drops | 1,4 | 0,5 | 24,1 | 3,5 |
| Salbutamol 100mcg inhalant | 1,6 | 0,3 | 37,9 | 1,9 |
| Simvastatin 10mg tablets | 1,5 | 0,3 | 30,8 | 1,9 |
| Sodium chloride 0,9% infusion | 1,1 | 0,3 | 11,3 | 2,2 |
| Tetanus vaccine injection | 1,5 | 0,4 | 28,6 | 2,4 |
| Tenofovir, lamivudine & dolutegravir 300, 300 & 50mg tablets | 1,5 | 0,7 | 33,7 | 4,7 |
| Tramadol 50mg capsules | 1,5 | 0,4 | 29,4 | 2,4 |

**Table S7.** Overstocking levels per facility

|  | | Overstocking (Units) log_10_ | |
| --- | --- | --- | --- |
|  |  | *Mean* | *Standard Deviation* |
| Facility | FAC-001 | 2.4 | 0.7 |
|  | FAC-002 | 2.4 | 0.8 |
|  | FAC-003 | 2.5 | 0.8 |
|  | FAC-004 | 2.6 | 0.7 |
|  | FAC-005 | 2.3 | 0.9 |
|  | FAC-006 | 2.2 | 0.9 |
|  | FAC-007 | 1.6 | 1.0 |
|  | FAC-008 | 2.4 | 0.8 |
|  | **Total** | **2.4** | **0.8** |

*Note.* Values were log-transformed.

**Table S8.** Value of expired medicines per facility

|  | | Value of expired medicines (ZAR) log_10_ | |
| --- | --- | --- | --- |
|  |  | Mean | Standard Deviation |
| Facility | FAC-001 | 2.9 | 1.5 |
|  | FAC-002 | 2.5 | 0.6 |
|  | FAC-003 | 2.8 | 0.6 |
|  | FAC-004 | 2.7 | 0.8 |
|  | FAC-005 | 2.8 | 1.2 |
|  | FAC-006 | 2.9 | 0.8 |
|  | FAC-007 | 3.2 | 0.9 |
|  | FAC-008 | 2.8 | 0.8 |
|  | **Total** | **2.8** | **0.9** |

*Note.* The values of expired medicines were log-transformed

**Figure S1** Simple graph for overstocking levels by medicines


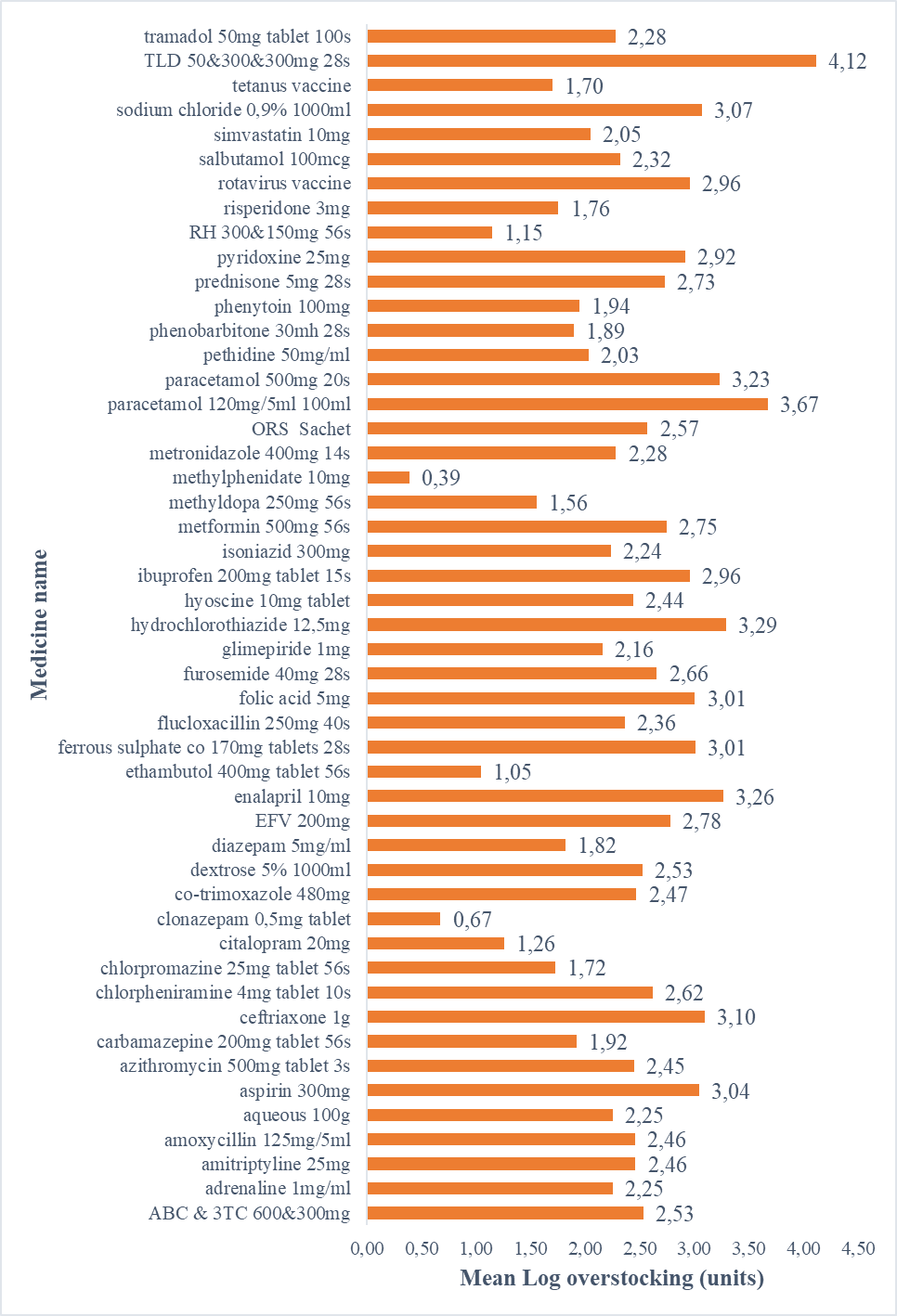

Supplement: Supplementary file 1 — Supplementary Material 1 [file 12913_2023_10096_MOESM1_ESM.docx]
